# Sitting by the Fire: Dene Perspectives on Indigenous Traditional Ecological Knowledges, Land Stewardship, and Community Wellbeing

**DOI:** 10.3390/ijerph23060716

**Published:** 2026-05-27

**Authors:** Danya Carroll, Jennie Vandermeer, Dëneze Nakehk’o, John B. Zoe, Celine Mackenzie Vukson, Nicole Redvers

**Affiliations:** 1 Schulich School of Medicine and Dentistry, Western University, London, ON N6G 2M1, Canada; dcarro4@uwo.ca; 2 Department of Indigenous Education, University of Victoria, Victoria, BC V8W 2Y2, Canada; 3 Łíídlı̨ı̨ Kų́ę́ First Nation, Fort Simpson, NT X0E 0N0, Canada; 4 Institute for Circumpolar Health Research, Yellowknife, NT X1A 3X7, Canada; 5 Chanie Wenjack School for Indigenous Studies, Trent University, Peterborough, ON K9L 0G2, Canada

**Keywords:** Indigenous knowledge, traditional ecological knowledge, Northwest Territories, Dene, Indigenous Peoples, climate change, Indigenous Land stewardship, Indigenous health, circumpolar

## Abstract

**Highlights:**

**Public health relevance—How does this work relate to a public health issue?**

- This work addresses notable areas of Indigenous health and wellbeing including Indigenous data sovereignty, land and environment, and climate change impacts on community health. Our work accentuates the interconnectedness between these areas and the holistic worldviews that Indigenous Peoples value.
- This work contributes firsthand community knowledge on the holistic health impacts (i.e., emotional, spiritual, mental, physical) that northern sub-Arctic Indigenous communities continue to experience due to changing environments and climate change.

**Public health significance—Why is this work of significance to public health?**

- This work centres Indigenous voices and perspectives on how northern Indigenous communities continue to protect and steward their health-related traditional ecological knowledges through processes that promote health and cultural continuity.
- Important aspects of Indigenous data sovereignty are explored in our research including gap areas and Indigenous community capacity to protect health-related knowledge. Indigenous data sovereignty is vital to Indigenous public health as Indigenous Peoples continue to seek ways to further protect their data and knowledge systems.

**Public health implications—What are the key implications or messages for practitioners, policy makers and/or researchers in public health?**

- Northern Indigenous communities are on the front lines because they are experiencing immediate and long-term impacts of climate change including on their lands, environments, food systems, health care systems, and ways of life.
- Our article platforms the stewardship role of Indigenous Peoples; Indigenous voices and leadership are needed in many policy and research spaces as we all continue to face multiple environmental crises (i.e., biodiversity loss, climate change, and land degradation).

**Abstract:**

Indigenous Peoples continue to steward their Lands through their traditional ecological knowledge (TEK), their Laws, and their kinship-driven processes as they have for millennia. There are various factors, including climate change, that threaten Indigenous TEK, Lands, and other processes including intergenerational knowledge transfer. Our team carried out a qualitative research study with Indigenous community members to increase understanding of Dene Peoples’ connections with Land, community TEK protection and stewardship, as well as changes in local environments. Semi-structured interviews were conducted with ten participants from the Northwest Territories (NWT), Canada, from December 2024 to February 2025. Coding and reflexive thematic analysis were carried out using qualitative software. Six themes were characterized from the interview data including: (1) intergenerational TEK are central to our ways of life; (2) despite various factors, our communities continue to share TEK across generations; (3) our collective health and healing are tied to our TEK as well as our values; (4) climate change-related threats and damages are impacting our People and the Land; (5) protecting and governing our own data is crucial for preserving our stories and knowledge; and (6) we need to protect Mother Earth for future generations. This study further demonstrates that the protection of Indigenous TEK is deeply important for the overall health and wellbeing of Indigenous Peoples. Additionally, the honouring of Indigenous sovereignty and Land rights is essential to transform current climate change approaches.

## 1. Introduction

Indigenous Peoples have maintained interconnected relationships with their traditional Lands and waterways for millennia. Through their long-standing values-based knowledge systems, Indigenous Peoples have effectively stewarded their territories, resulting in unimpeded, biodiverse, and intact ecosystems [1,2,3]. There is also a broad global understanding amongst many Indigenous Peoples that without healthy and ecologically intact Lands, the health and wellbeing of Indigenous Peoples is undermined [4,5]. Therefore, environmental stewardship is often prioritized. With multiple and colliding crises happening globally, the ability of Indigenous Peoples to continue to steward their Lands, is being increasingly challenged. With ongoing pressures from climate change as well as land grabs or forced land evictions (physically or culturally) for resource extraction and mining, agriculture, urban sprawl, fortress conservation, and other development initiatives (e.g., hydroelectric dams), Indigenous Peoples are often on the front lines of Land destruction. Yet, in tandem, Indigenous Peoples and their knowledges are increasingly being looked to for solutions to the climate and biodiversity crises [6]. This includes Indigenous traditional ecological knowledges (TEK), with its special emphasis on the relational connection to Lands and Waters for long-term environmental stewardship.

Due to the incredible diversity among Indigenous Peoples across geopolitical regions and cultures, there is no universal definition of TEK. In the United Nations system body, there is also no official definition of Indigenous Peoples for many complex reasons. Canada, however, recognizes three distinct groups of Indigenous Peoples, including First Nations, Inuit, and Métis Peoples, that have their own distinct languages, cultural practices and histories [7]. Ultimately, each Indigenous community has its own way of describing TEK through its Indigenous languages and cultural practices. For the purpose of this paper, however, we are defining traditional ecological knowledges broadly as dynamic, holistic, action- and place-based knowledges that are rooted in “millennia of witness” and kincentric relationships with all living beings (i.e., plants, animals, water, etc.) [8,9]. We specify a broad definition of TEK for the purposes of binding our narrative to a common understanding yet acknowledge and make space for the diverse views and expressions of TEK within and throughout Indigenous communities globally.

Indigenous Peoples’ knowledges, inclusive of TEK, have withstood many attempts by colonial governments and institutions to eliminate them. Across Turtle Island (i.e., North America), through government-led policy efforts, Indigenous children were forcibly taken to residential schools (Canada) and boarding schools (United States [US]) [10,11], where colonial education systems and epistemologies were violently forced on them, resulting in substantial impacts on overall health and wellbeing [12]. During this time, Indigenous knowledges, languages, and ceremonies were suppressed in North America through policies such as the Indian Act in Canada [13] and the Dawes Act in the US [14]. These policies created a disruption in cultural education and a forced disconnect between children and their communities, including with Elders [15]. For example, First Nations potlatch and Sun Dance ceremonies were banned in Canada by a provision in the Indian Act [15]. During the period when many Indigenous cultural practices were banned, Indigenous communities often attempted to maintain their cultural and spiritual traditions through underground practice, sometimes risking imprisonment to ensure the survival and transmission of these vital practices and knowledges for future generations [15]. Regardless, there were still significant disruptions to intergenerational knowledge transfer between Indigenous communities and their children, resulting in valuable TEK being diminished or in some cases lost [16]. With this, the impacts of colonization continue to be felt by Indigenous Peoples today, sometimes expressed through intergenerational trauma as well as impacts to overall health and wellbeing [17,18]. Despite ongoing challenges, Indigenous Peoples continue to work towards preserving and revitalizing their knowledges and stewardship roles.

Traditional ecological knowledges and practices are also essential to the health, wellbeing, and survival of many Indigenous Peoples. The essential nature of TEK is amplified in circumpolar regions such as the Canadian sub-Arctic. Northern Canadian landscapes are crucial as they contain 20% of the world’s freshwater resources [19] and a large portion of boreal forests that regulate climate and store carbon [20]. Yet, northern Canadian lands and waterways are under constant threat from climate change as the region is experiencing warming at a rate three to four times faster than the global average [21]. This includes the Northwest Territories (NWT), home to three recognized Indigenous Peoples, including First Nations, Métis, and Inuit Peoples, who are on the front lines of climate change as well as destructive mining activities. Increasing temperatures are having substantial impacts on NWT waterways (e.g., droughts) which affects culturally important lakes and rivers, and also ramps up wildfire activity that ultimately impacts Indigenous communities’ food sovereignty, transport and land travel, as well as livelihoods [22].

Many Dene First Nations Peoples living in the NWT continue to rely on the Land for food, economic, medicinal, and cultural purposes. Subsistence lifestyles carried out through TEK, including hunting, fishing and trapping, are still actively practised in many communities. Many of these practices, such as subsistence hunting, are impacted by various factors including concerns about environmental contamination and climate change [23]. Despite ongoing environmental impacts, Dene Peoples throughout the NWT are vital to climate change, conservation, and environmental discourse and practice due to their extensive knowledge of their Lands and Waters. Their TEK is important for adapting to and managing the challenges posed by rapid, unanticipated environmental and climate change shifts [24]. Dene Peoples are also key contributors to protecting various culturally significant species including those that are becoming increasingly threatened, such as caribou [25]. They are also crucial in providing long-term monitoring and culturally based observations of changes to local Lands, environments, and biodiversity [24]. For example, Dene Peoples and their TEK are crucial to monitoring northern Land-based food sources and in co-leading development of adaptation strategies for food security [26].

Euro-Western research institutions have been documenting the changes that Indigenous Peoples have been observing for many decades in the north—yet research processes have not consistently included meaningful collaboration with Indigenous Peoples or their leadership. These institutions have also increasingly sought to incorporate or integrate Indigenous knowledges into Euro-Western research frameworks. Misguided attempts by researchers to integrate Indigenous knowledge, however, have often missed crucial components, including the context of Indigenous worldviews, values, and governance [27]. This has led Indigenous scholars to highlight that Indigenous Peoples’ TEK cannot just be selectively integrated into Euro-Western frameworks [28]. Doing so risks ongoing cultural appropriation and/or misuse of TEK while disregarding the relational worldviews and values that encapsulate and underpin TEK [28]. These risks of misuse are further amplified at larger scales of influence, such as in the nature-based solutions and climate change space, where policy and practice are often divorced from the recognition of Indigenous voices, rights, and governance [29]. Positive movement is happening in some regions with the inclusion of Indigenous TEK in schools and universities, including in research-related studies and curriculum [30]. Concerns remain, however, regarding how to respectfully work with Indigenous Peoples and their TEK, particularly in institutional settings, given increasing awareness around Indigenous knowledge ownership and protection of Indigenous data.

Indigenous data sovereignty is additionally becoming increasingly important for Indigenous Peoples as they seek further ways to protect their data, including TEK data, through ownership, access, and storage mechanisms [31]. Many Indigenous communities continue to pass on orally transmitted knowledge while also recognizing the need to store and preserve TEK more formally through physical or digital repositories [32]. Indigenous repositories are physical and/or digital spaces where Indigenous data is housed under the direct oversight of Indigenous Peoples. There are considerable challenges that exist for Indigenous communities who want to store and protect their data, including increasingly having to deal with frequent climate-driven changes [33] and cascading disasters such as forest fires and flooding. For example, in 2023, the NWT experienced an unprecedented wildfire season that substantially impacted Indigenous communities [34]. These wildfires resulted in the loss of valuable Land areas as well as the destruction of the Yamozha K’ue Society repository building which housed many items that were culturally significant to Dene Peoples [35]. As northern Indigenous communities continue to confront substantial changes in the Land and climate, there is increased recognition of the need to further secure and protect their Indigenous TEK. There may be challenges, however, in the capacity and support available for Indigenous communities to be able to do this. With this, there is a lack of overall Indigenous-led research on how Indigenous communities perceive and adjust to climate change-related challenges regarding TEK transmission and storage in the context of health and wellbeing.

Responsive to community calls for greater Indigenous-led TEK research, we aimed in this qualitative study to explore Dene perspectives from the NWT, Canada, on health-related Indigenous traditional ecological knowledge stewardship and protection in the region, inclusive of observed local changes to the Land. We also sought to gain insights on health-related TEK data protection from external threats including climate change.

### Positionality

It is increasingly expected that researchers conducting research in Indigenous communities, including research related to Indigenous epistemologies and ontologies, share their positionalities [36,37]. As Indigenous authors from varied communities, we share our positionalities here. The first author is an Indigenous community scholar from the Diné and White Mountain Apache Tribal Nations in the southwestern US. The second author (JV) is Sahtúgot’ı̨nę Dene from Délı̨nę, NWT, a Dene Ked$\overset{´}{ǝ}$ language speaker, and an Indigenous graduate student in Indigenous Language Revitalization. The third author (DN) is a proud member of the Łíídlı̨ı̨ K$\overset{´}{ų}\overset{´}{ę}$ First Nation living and working in *Denendeh* (Land of the People), or the NWT. The fourth author (CMV) is from the Tłı̨chǫ region in the NWT and is an Indigenous PhD student in Indigenous Studies. The fifth author is an Indigenous knowledge holder also from the Tłı̨chǫ region of the NWT. The last author is an Indigenous and planetary health scholar from the Deninu K’ue First Nation, also located in the NWT.

## 2. Materials and Methods

### 2.1. Research Questions

This research study was led and conducted by an all-Indigenous research team, including team members that are from the communities where the research was carried out. Research questions for this study included: (1) What are the local environmental and climate changes being observed by Indigenous communities in the NWT that are affecting the protection of health-related TEK? (2) How do NWT-based Indigenous communities steward and protect their health-related TEK? (3) What are the gaps in NWT-based Indigenous community-level protection of Indigenous TEK amidst environmental and climate changes?

### 2.2. Overall Study Design

This qualitative research study was grounded in an Indigenous relational conceptual framework that aimed to honour the Indigenous communities that participants represented as well as their respective kinship structures [38]. Our framework recognized the “inter-Indigenous relationships” between participants and researchers, as we all come from different Indigenous communities but ultimately created shared spaces where we brought our Peoples’ ways of knowing together [39]. Through the relational conceptual framework, we aimed to create a virtual space with an overarching decolonial approach. Relational-based Indigenous frameworks and methods are crucial for bringing culturally driven research to life. Our research study received ethics approval from the Western University Non-Medical Research Ethics Board (#125143) and the Government of the Northwest Territories (NWT Scientific Research Licence No. 17603). This study followed the Standards for Reporting Qualitative Research (SRQR) (see Supplementary File S3) [40].

### 2.3. Setting

The research was conducted virtually using Zoom and involved participants from communities located across the NWT, Canada (making up part of the Circumpolar North; see map here). Some of the communities are only accessible by boat or plane and not by road. The NWT population is estimated to be around 45,000, of which over half self-identify as First Nations, Métis, or Inuit Indigenous Peoples [41]. Nine Indigenous languages are officially recognized in the NWT, including many Dene First Nations’ language dialects [42]. Dene Peoples, who make up the majority of the Indigenous population in the NWT region, often refer to the Land area they have inhabited since time immemorial as *Denendeh* [43]. There is a mix of regional and community-based Indigenous governments in the NWT that act as significant co-leaders in Land and water stewardship in the region [44]. Indigenous Peoples therefore continue to honour and uphold their relationships with the Land, water, and non-human relatives in the region.

### 2.4. Recruitment and Consent

For this study, Indigenous community members were eligible to participate if they were 18 years or older. Potential participants were identified through existing networks known to one of the researchers, with consideration made for geographic, gender, age, and leadership representation (i.e., from conservation and TEK backgrounds). This approach was helpful as Indigenous communities in this region are widespread and may be challenging to access for those not familiar with community protocols. Potential participants were contacted by email to determine their interest in research participation. If a potential participant was interested, the research team provided them with information on the study and gave them time to consider and ask any remaining questions. Potential participants were verbally consented to via Zoom by a research team member (DC) (see Supplementary File S2). Per Indigenous-based protocol in the region (both research and traditional protocol), each participant was provided an honorarium for sharing their time and knowledge, as well as a traditional offering. No participants declined participation or withdrew from the research study. 

### 2.5. Interview Data Collection

Ten semi-structured interviews were conducted in English by a female research team member from December 2024 to February 2025. There are ongoing debates about appropriate sample size in qualitative research, often stemming from quantitative or positivist orientations. From an Indigenous research methodological orientation, the depth and comprehensiveness of the data are balanced with Indigenous member checking to ensure sufficient data for appropriate analysis relevant to the research questions posed. Accordingly, ten participants provided the depth and range of data needed for this study’s objectives. The interviews were audio recorded and lasted between 30 min and 2 h. The semi-structured interviews allowed for high flexibility in sharing and listening between the participants and the researcher, as well as fostered spaces for storytelling. Ensuring space for storytelling provided a critical lens that promoted reflection and new understandings between the Indigenous researchers and community-based participants [45]. The interview guide included questions on connections to Land, stewardship, and the protection of Indigenous TEK, as well as questions on noticed changes to the Land and local environment (see Supplementary File S1).

### 2.6. Data Analysis

The interviews were transcribed verbatim using the NVivo Transcription (15.2.2.(13)) software. A research team member (DC) then reviewed and verified the interview transcripts to ensure accuracy. The transcripts were then deidentified and uploaded to the NVivo software (version 15.2.2 (13)) for qualitative data analysis. Reflexive thematic analysis (TA) was used as outlined by Braun & Clarke [46] to support the characterization of key themes from the data. Using a reflexive TA supported a more flexible and fluid approach to exploring the interview data. This also allowed us to include narrative elements from storytelling that might be overlooked by other research methods. Preliminary inductive coding of the transcripts was carried out by one author (DC), with a second author (NR) brought in for refinement of the codes and theme development, as well as for auditing purposes. Member checking was done with a few participants to ensure the interpretation of their quotes was accurate.

## 3. Results

Ten semi-structured virtual interviews were conducted with participants between December 2024 and February 2025. All participants in the study identified as Indigenous, with six identifying as female and four as male; however, no notable differences were noted between genders in the results. The age range of the participants was from 39 to 74 years of age, with the average age of the participants being 60 years old. Six themes were characterized from the interview data through reflexive TA (see Table 1) and are further discussed below.

### 3.1. Intergenerational TEK Are Central to Our Ways of Life

Throughout the interviews, participants shared their perspectives on the close relationships their Peoples have had with the Land since time immemorial. Several participants described how their Peoples have accumulated vast TEK based on their relationships with the Land. One Elder participant shared the following:

*and they [the People] know the territory so well. They know where the trails are. They know where the trap lines are … they used the Land for medicine, the plants for medicine. And they know where the fish are.*

(CAN010)

Participants noted how significant it was for their families and communities to be able to have opportunities to access TEK-related teachings on the Land. These opportunities were noted to occur naturally (i.e., within kinship structures) or to be part of more organized activities such as on-the-Land programming. One Elder participant stated the following:

*[a] way to keep people on the land is to make sure that the … information of the land is always made available and … are being kept up and made strong by collective knowledge … and mapping those things out [the knowledge and information] and making them available and making it part of curriculum.*

(CAN008)

Participants also discussed how various aspects of TEK continue to be shared. For example, several participants described how the intergenerational transfer of TEK occurs on the Land (subtheme) and why this continues to be a pivotal approach to protecting their TEK. TEK being shared directly on the Land itself was therefore broadly seen by participants to be the ideal place for intergenerational knowledge transfer to occur.

*Elders are telling stories while they’re on the land, and it comes alive for us … I think we haven’t tapped into all the tools that they use. Sometimes I think about that when I’m on the land. What else is out there that we don’t know about?*

(CAN004)

Many of the participants also reiterated how their families and communities learn key life skills on the Land beyond just survival. Some participants noted how knowledge is also shared in the form of stories. For example, this would often happen around a campfire where everyone may be gathered.

*[T]he transfer of knowledge really happened over the campfire. [A]nd, you know, you’re going to hear a good story or … someone is going to share something with you.*

(CAN003)

There was discussion on how TEK are transmitted on the Land through local Indigenous languages. Language is an essential vessel to protect our TEK (subtheme) emerged as a key area of discussion with some of the participants. Some participants noted that Indigenous languages are essential to the sharing and protection of TEK.

*I think what is also very, very important is the language. We need … [it] in order for us to protect our knowledge, [and] traditional system. We need to protect our language.*

(CAN004)

Along similar lines, the revitalization of local Indigenous languages was discussed by some participants. One participant shared some of the work being done in their community around language revitalization relevant to TEK:

*I’ve been really open now to even exploring AI, and I’m involved with this project with Indigenous language revitalization, with my nation, on exploring AI to transcribe and translate in our [name] languages.*

(CAN010)

Although technology was seen to be helpful to some language revitalization processes, it was emphasized that caution is needed when considering the use of these tools. The oversight of local First Nations governments was seen as important to any bridging of Indigenous language learning and technology. Overall, the processes through which intergenerational transfer of TEK occurs were seen as equally important as the knowledge itself. To ensure these processes continue, it was emphasized by some participants that the protection of local Indigenous languages is also crucial. Overall, participants shared how crucial their Peoples’ TEK is to their way of life.

### 3.2. Despite Various Factors, Our Communities Continue to Share TEK Across Generations

Throughout the interviews, participants shared how historical impacts, including colonization, have led to collective trauma that continues to affect their communities. These impacts were broadly discussed by participants as also affecting the transfer of TEK within their communities. One participant stated the following:

*[t]here’s a lot of that trauma … that’s not our fault, but a lot of our people carry it … that’s a big barrier and a challenge for continuing on those things [intergenerational transfer] and carrying the stories … [T]he only thing we can do is to try to continue to do those things, to try to see each other, to try to work with each other, to try to believe in each other.*

(CAN003)

Participants also commented that their communities have withstood many of the historical and colonial impacts throughout time and continue to embrace their knowledges. Some of the participants, for example, shared how they were residential school survivors [11] or children of survivors. It was therefore noted that despite deliberate attempts to eradicate their knowledges and ways of life, they have persisted. With this, it was highlighted that residential schools continue to impact our knowledge and wellbeing (subtheme), and that this must be acknowledged when discussing knowledge broadly beyond TEK. One Elder shared some insights on his experience, particularly after attending a residential school.

*I was seven years old when I went there [residential school] … So I lost a lot of friends … They didn’t learn how to survive this colonization, assimilation, loss of language, culture, you know, identity of who you are … And that’s what my late father … told me…* [Indigenous language words]*… That means residential school destroyed people.*

(CAN006)

Participants emphasized how this residential school period of time fractured their communities’ knowledge systems and disrupted connections to the Land and overall wellbeing. Some participants also shared how being descendants of residential school survivors has also had substantial impacts on the transfer of TEK. One participant spoke about their firsthand experiences.

*I think it’s that pain, that fracturing, that’s really had some negative consequences on how TEK is transferred between generations, because there’s just so much pain there … And I know what that’s like because … I work with my community, and these are the most … capable people on the Land and most intelligent people you’ll ever meet in your life. But if they’re so angry and bogged down and they don’t have the patience to teach you, then that knowledge is lost.*

(CAN005)

The lived experiences shared by participants demonstrate the ongoing impacts of residential schools on the protection of Indigenous TEK, including how TEK is shared.

### 3.3. Our Collective Health and Healing Are Tied to Our TEK as Well as Our Values

Several participants shared perspectives on how important collective healing from past trauma induced by colonization is for their communities. Healing holistically (i.e., emotional, spiritual, mental, and physical healing) was broadly seen as being most effective by being on, learning from, and having relationships with the Land. Some participants also emphasized that by continuing these close relationships with the Land it was also healing for the Land itself. One Elder participant shared that, *“[t]he ecological system is healing. It’s healing to our people … because of what we went through”* (CAN004).

Throughout the interviews, some participants also shared that their communities are increasingly going back onto the Land. They noted that there has been more movement by communities to continue their healing journeys on the Land. One participant discussed that some of the Land-based camps (i.e., a term used in this region that is similar to culture camps) are being hosted to support health and wellness within the community:

*In terms of … like wellness camps where they bring in Indigenous counsellors and they host these camps on the Land with families for healing and wellness.*

(CAN005)

Participants explained how vital these types of camps are to overall individual and collective healing within their communities. Our Land heals us holistically and spiritually (subtheme) was therefore a key point of discussion. Some participants, for example, shared their personal experiences of being on the Land and the sense of healing it brought to them. One participant shared, *“[t]he land is healing. It heal[ed] me* [to] *who I am today”* (CAN002). With this, an overall common narrative shared by participants during the interviews was that the Land is necessary to their communities’ overall health and wellbeing.

Additionally, it was expressed by participants that the Land is a sacred place for learning and teaching. The Land continues to be our sacred teacher (subtheme) was therefore highlighted, with several participants sharing their experiences of Land-based education.

*And today in this community there’s lots of traditional people … there’s a lot of traditional hunters … people go hunting. You go trapping, go fishing. We have those kinds of people … It’s just like one semester. Teach them how to live off the land, how to respect the land. Teach them the history, language.*

(CAN006)

In summary, participants shared an overarching viewpoint that being on the Land was vital to community wellbeing, a sense of connectedness, and an overall important way of life. One participant pointed out how the Land brings the community together: *“And so when they’re travelling the landscape together, paddling, they get to hear the language, get to see the land, … get to see a lot of the things that I mentioned. And it also makes people work together”* (CAN009).

### 3.4. Climate-Related Threats and Damages Are Impacting Our People and the Land

Given that all of the participants were from northern sub-Arctic communities, they provided considerable insight into the ongoing impacts of climate-related changes in the region. Participants shared some of their firsthand observations of how changes in the Land were directly impacting the safety of being on the Land. For example, one participant shared:

*We’re worried about the safety of the land, the integrity of the land, but not only how it impacts us, but how it’s impacting the water, how it’s impacting the air because its forest fires smoke, and how it’s impacting the animals.*

(CAN003)

Communities, through TEK, are observing Land changes in real time (subtheme), which was noted as a critical area of focus within the perspectives shared. Some Elder participants provided longer-term observations of Land changes. One Elder shared that, *“[i]t doesn’t get that cold no more. Yeah, my dad said* [it] *used to get so cold you can hear the trees cracking”* (CAN006). Participants also shared how unseasonably warmer winters have affected the ice needed for transportation. One participant stated:

*I’m worried that we’re not going to be able to travel on the river anymore. And that’s our main mode of transportation, because we don’t have an all-weather road in my region where [we] fly in, fly out. And so we have to wait for the winter road and our ice roads in the winter to get all of our gas and food for … the year.*

(CAN005)

The lived experiences shared by participants highlighted how climate change was having drastic impacts on their communities, including the ways that their Peoples share TEK. Climate change is restricting access to our Land and cultural education (subtheme) was also brought up by some participants. One participant noted that, *“[t]here* [are] *places we can’t go anymore. We can’t access those places because the water’s too low. We can’t travel in those places … It’s places where we harvest medicines”* (CAN003). The strong, close relationships with the Land were seen by participants as being especially critical during ongoing Land changes.

*Especially here in the north. Climate change is huge here…And because of our close relationship with the land, you really see it. You really feel it. You really experience it.*

(CAN008)

Throughout the interviews, participants provided powerful insights into how climate-related changes are impacting local ecosystems and landscapes. The extent and scale of these impacts, including the impacts on the communities’ relationships with the Land and what that could mean for overall community health and wellbeing were seen as concerning.

### 3.5. Protecting and Governing Our Own Data Is Crucial for Preserving Our Stories and Knowledges

When participants were asked about community-level protection of data, including TEK, they emphasized the importance of protecting their Peoples’ stories and narratives. One participant asserted that, *“[w]e need more control over our own information, but we also have to be in charge of our own narrative”* (CAN003). Although not explicit, some participants shared how their knowledge should lead the way in how data protection processes occur. One participant noted that *“[i]t’s important to invert that dynamic and have Indigenous knowledge be like the leading framework”* (CAN007). Some participants also shared details of current data repositories in their communities. For example, one participant described her community’s data infrastructure:

*My community has what they call [name] cultural commons. And it was started a few years ago, and it’s operated by [name] government. They control the digital archives, and anything that’s relating to the research of our content, culture, way of life, and language and stories of our [name] people, they are recorded, saved in their … archives.*

(CAN004)

The importance of community-based data repositories was seen differently in each community context, with some participants sharing that some of their community’s data and materials are already housed. Some other participants expressed concerns around the need for more stable data storage processes and capacity, including the need to strengthen TEK data storage and protection in their communities. With this, enhancing the storage of our TEK data is necessary (subtheme) was discussed as being crucial in a time of increasing climate change-related events such as forest fires and flooding. One participant shared the progress that has been seen in a community they have worked with in moving from cassette tape forms of storage to digital formats:

*When I started 20 years ago, it [i.e., TEK data] was all like in tape form. But since I started working and we started focusing on trying to keep everything in-house and then giving copies to them [i.e., outside entities] rather than giving them everything. Yeah, we started doing copies. First was on CDs and then USB drives, and then it just evolved. Now it’s just like a downloaded link.*

(CAN001)

Overall, throughout the interviews, participants expressed the need for more support in building out their communities’ capacity to implement data storage processes.

### 3.6. We Need to Protect Mother Earth for Future Generations

The role of Indigenous Peoples as the original stewards of the Land was emphasized by participants in the interviews. There was also discussion on how their TEK is especially needed in contemporary contexts. One participant encapsulated the importance of this stewardship responsibility:

*As [name] and what my role is in continuing to take care of the land … [I] think it’s a very high responsibility. … And it’s been instilled in me my whole life that it’s our job to take care of the land because we come from it. And that’s our role … Sharing our TEK with the world in a way that is respectful and engaging and really meaningful and authentic is what the world needs. They* [non-Indigenous Peoples] *need a different perspective. They* [non-Indigenous Peoples] *need a different way of looking at the world because, like I said, their way is just not working.*

(CAN005)

The broader application of TEK was emphasized by some participants when stating how valuable their role is for environmental stewardship in the NWT. Some participants also shared that they would like to see their communities continue to expand their environmental stewardship roles.

Indigenous Land management and conservation are thriving in the north (subtheme) was characterized as an area of discussion by some participants. One participant shared how they see the stewardship role of Indigenous Peoples becoming stronger and more needed.

*The growth of work like Guardians programming. So getting our own people out there on the land, doing work on behalf of our governments to gather information, be a presence on the land. You know, the growth of the Guardians program across the north has really strengthened over… particularly the last number of years. Indigenous, protected and conserved … areas generally are sort of an implementation of the vision around land use planning.*

(CAN007)

Participants also shared that their role as environmental stewards and Guardians is especially important because of the ongoing constant threats to the Land from resource extraction and mining industries. Our collective responsibility is to protect our Land and Peoples from resource extraction (subtheme) was therefore shared by some participants who have seen extractive activities happening firsthand in their communities. For example, there were concerns shared by participants about the ongoing effects on the Land and their Peoples from mining and the Alberta oil sands operations. Participants expressed the importance of continuing to speak up and advocate for the care and protection of the Land and water. There was also a broad viewpoint on the need for more support and recognition from outside entities of Indigenous TEK in different spaces, including in leadership spaces. Collectively, participants shared a viewpoint of their key roles as stewards, which was articulated by one participant as, *“The Elders always said ‘Protect your land, fight for it, don’t give up. Work together, stand up together.’”* (CAN010)

## 4. Discussion

*In order to be … healthy, thriving people, we need to take care of the areas* [i.e., the Land] *that help make us healthy, thriving people.*

(CAN007)

Various Indigenous perspectives from the NWT, Canada, on TEK were captured throughout our qualitative research study. Participants shared how their communities steward and protect their TEK, Lands, and ways of life. Specifically, we characterized six themes from the interviews carried out, including: (1) intergenerational TEK are central to our ways of life; (2) despite various factors, our communities continue to share TEK across generations; (3) our collective health and healing are tied to our TEK as well as our values; (4) climate change-related threats and damages are impacting our People and the Land; (5) protecting and governing our own data is crucial for preserving our stories and knowledge; and (6) we need to protect Mother Earth for future generations. The knowledge and perspectives shared by participants highlighted overarching key concerns around TEK data protection, Land stewardship, intergenerational knowledge transfer, and collective wellbeing. Participants in this study viewed Indigenous TEK and data protection from a holistic standpoint.

This study also further demonstrated how Indigenous Peoples are leading environmental stewardship in their communities throughout northern Canada. Indigenous Land Guardian programmes that prioritize TEK are not a new concept to some Indigenous communities, with many leading in this space [47,48], including through youth programming [49]. Indigenous TEK has been seen to be vital to maintaining healthy and balanced ecosystems and Lands worldwide [50]. Indigenous Land Guardians (Canada) and Rangers (Australia) are examples of Indigenous-led stewardship programming that have beneficial impacts for Indigenous Lands and Peoples [51,52]. There are also broader opportunities for Indigenous co-management and collaboration with local communities including those that work in urban [53] and recreational Land spaces [54]. The Indigenous Land Guardian and Ranger programmes may be helpful models for other Indigenous communities that are working towards strengthening their stewardship roles and collaborative processes [55]. Indigenous Land stewardship programmes are vital for protecting Land as well as promoting Indigenous health and wellbeing [56].

This study additionally aligns with broader understandings and research affirming how Indigenous Peoples continue to steward Land and freshwater [57] as well as safeguard most of the world’s remaining biodiversity [58] through their TEK. In Canada, there is increasing movement and support for Indigenous environmental stewardship including through Indigenous protected and conservation areas [59] which account for 1% of Canada’s landmass [60]. Participants in this study come from Indigenous Nations that are also leading in Land and biodiversity stewardship spaces in the NWT. These Indigenous Nations, similar to many Indigenous Peoples across the globe, are vital examples of exemplary ethical communities that embody sustainability strategies that have a millennia-old track record [61]. For example, Thaidene Nëné is an Indigenous protected area on the homelands of the Łutsël K’é Dene First Nation where their stewardship follows Dënesųłıné values and Laws (see Box 1) [62]. Furthermore, the “NWT: Our Land for the Future” initiative is currently one of the largest Indigenous-led land conservation initiatives globally, in which 21 local Indigenous governments and partners aim to conserve and steward up to 380,000 square km of Land and inland water by 2030 [63].

Box 1 **Indigenous Stewardship in the: Thaidene Nëné.**

In 2019, Thaidene Nëné was established as an Indigenous protected area by the Łutsël K’é Dene First Nation in the NWT, Canada. Thaidene Nëné consists of 6.5 million acres that include boreal forest, tundra and the east arm of the Great Slave Lake (deepest freshwater lake in North America). Łutsël K’é Dene Peoples rely on their Land and the Lake for health, sustenance, transport and economic purposes as they are a remote community. The Łutsël K’é Dene First Nation co-manages portions of Thaidene Nëné with the Government of the NWT and Parks Canada. These portions have been designated as a territorial protected area, wildlife conservation area, and national park reserve. This historic unique partnership marks a commitment between an Indigenous First Nation, territorial, and federal governments to protect and steward vital natural and cultural resources as well as promote sustainable economic livelihoods (e.g., fishing, trapping). This important partnership, which prioritizes Indigenous Land stewardship and governance through TEK-based approaches, can serve as a model for the globe.

Dene Peoples are also actively engaging in collaborative northern-based research which can support the decolonization of colonial conservation approaches [64]. Some collaborative research frameworks have been effective in bridging Indigenous TEK with Western science, including studies on caribou species variation [25] and conservation planning [65], all known to have direct impacts on Indigenous health and wellbeing [66,67]. These kinds of collaborative research projects demonstrate that research is more effective when it is co-developed with Indigenous Peoples and includes the prioritization of culturally significant metrics [25]. While Indigenous communities in the Canadian north continue their environmental stewardship roles, Indigenous Land rights continue to be disregarded in many settings including through land grabs and forced land eviction [68]. It is important to note that Indigenous communities are diverse, and some communities may have limited or no access to their Lands from which their TEK emerges [69,70]. There continues to be a high need for increased recognition and support for Indigenous-led Land stewardship programmes around the globe, including for policy-driven initiatives that recognize Indigenous Land rights and governance as being inherent to Indigenous health and wellbeing as well as overall planetary health [4].

This study further highlights the importance of supporting Indigenous Peoples themselves as they work to further protect their own TEK. Indigenous Peoples and their TEK continue to provide valuable contributions to the ongoing climate change and planetary health discourse. Furthermore, there is a need for more emphasis on the holistic approaches that Indigenous TEK can offer to environmental sustainability and planetary boundary dialogues amid the Homogenocene (i.e., the current time period identified by the Euro-Western scientific community which is marked by rampant human impacts on Earth’s biodiversity). As Indigenous Peoples continue to be impacted by climate-driven changes, these changes are also exacerbating Indigenous health inequities [71] and directly threatening Indigenous medicine systems [9]. Climate change also continues to threaten Indigenous TEK, Lands, and wellbeing across the globe [72,73]. Although Indigenous Peoples’ TEK is often sought after for inclusion in climate change spaces, there is a continued “unwillingness to recognize Indigenous jurisdiction and Indigenous understandings of land as systems of reciprocal relations” [74] which can perpetuate TEK extraction. Furthermore, collective harm imposed on Indigenous Peoples and their Lands continues to happen globally as they continue to be pressured by state governments for resource extractive [75,76] and colonial conservation projects [77]. These violations affect the ability of Indigenous Peoples to fully contribute to the overall health and sustainability of the planet. Indigenous Peoples must be engaged with on equal terms including through free, prior, and informed consent (FPIC) and meaningful consultative processes. The United Nations Declaration on the Rights of Indigenous Peoples (UNDRIP) states that FPIC is crucial to protecting and upholding the rights of Indigenous Peoples [78]. Ultimately, Indigenous Peoples and their knowledge are necessary for transforming climate and planetary health spaces.

Explicitly and implicitly, Land-based healing through intergenerational transfer of TEK, and how this process contributes to overall Indigenous health and wellbeing were repeatedly highlighted in our findings. Land-based healing is rooted in the Indigenous worldview, identity, and the relationship that comes from being on the Land [79]. There is no universal definition for Land-based healing; however, there are similar shared principles among Indigenous Peoples including the importance of intergenerational sharing, honouring interconnectedness among all living beings, and upholding cultural protocols and practices [80]. Cultural continuity and intergenerational transfer are essential processes that occur in Land-based healing that also support the protection of Indigenous TEK. These processes also catalyze the uptake of cultural protective factors that are paramount for protecting and supporting Indigenous health [81,82,83]. Land-based programming is a holistic approach that is being embraced and implemented by Indigenous communities to increase access to TEK for Indigenous youth [84] and to promote safe healing spaces [79]. These programmes are vital to many areas of Indigenous health including mental health [85,86] and youth development [87]. There is a common viewpoint among Indigenous Peoples across North America [88,89] and beyond [90,91] that the Land and Indigenous TEK is significant for their overall health and wellbeing.

Overall, this research study highlighted that there continues to be a need for increased support for Indigenous Peoples to effectively mitigate the multiple compounding factors negatively impacting their TEK, Lands, and wellbeing. This support is vital to preventing TEK extraction and must platform Indigenous worldviews and knowledges as well as respect the Land rights of the original stewards and caretakers of these Lands. Moreover, rights-based approaches that position Indigenous Peoples as “rightsholders rather than stakeholders” can also further align with policy frameworks including UNDRIP, the Paris Agreement, and the Convention on Biodiversity Decision 16/19 [71]. Future research is needed to further examine the factors that encompass Indigenous TEK protection including repository and internal community capacity development (e.g., training, personnel), and the types of support (e.g., funding, resources) needed to operationalize these processes.

### Limitations and Strengths

This qualitative study was carried out in a virtual setting with participants located in various Indigenous communities in the Canadian sub-Arctic. These Indigenous communities have their own unique knowledge systems, so the transferability of this study to other contexts may be limited. Despite this, there are noted similar experiences and impacts being felt by other Indigenous communities as reported in the literature above. Broad themes and issues may therefore be relevant to other contexts. More specifically, participants shared viewpoints around knowledge, Land, and environmental stewardship that may be viewed similarly by other Indigenous communities.

Additionally, it must be highlighted that community-based participants were included in this study; however, they may not be representative of the entire community’s viewpoints including across ages, genders and geographic areas. We made efforts, however, to ensure representation of varied genders, age groups, and geographic areas in the north, including from known knowledge holders, which gives us more confidence that the findings are relevant for the context where the research was carried out. Given this, our study may further inform local and regional community planning.

## 5. Conclusions

This Indigenous-led research study provided insights and perspectives on the ways Indigenous communities in the Canadian north view, honour, and protect their TEK. The participants emphasized the importance of the interconnectedness between TEK, the health of the Land and themselves. The protection of Indigenous TEK was seen to be possible through Indigenous-led Land stewardship approaches that are additionally important for overall Indigenous health and wellbeing. Honouring Indigenous Land rights and the sovereignty of Indigenous Peoples are therefore essential to improve and transform current climate change and conservation approaches to ensure Indigenous and overall planetary health.

Indigenous Peoples, especially those from circumpolar regions such as the NWT, are keenly aware of the various dire impacts of climate-driven changes; therefore, their TEK contributions must be at the forefront of climate action through Indigenous rights-based approaches, including free, prior, and informed consent. As noted by the title of this research study, which was highlighted by one of our participants, an invitation to sit by the fire to sit and share embodies reciprocal respect but also inherent responsibilities. Continued TEK extraction, either through research or practice, cannot continue. Instead, shared ethical spaces such as those invoked by Indigenous traditional protocols, including those occurring when sitting around a fire, need to be valued, appreciated, and ultimately respected.

## Figures and Tables

**Table 1:** Themes and subthemes from the semi-structured interviews.

Theme	Subtheme
Intergenerational TEK are central to our ways of life	- Intergenerational transfer of TEK occurs on the Land - Language is an essential vessel to protect our TEK
Despite various factors, our communities continue to share TEK across generations	- Residential schools impacted our knowledge and wellbeing
Our collective health and healing are tied to our TEK as well as our values	- Our Land heals us holistically and spiritually - The Land continues to be our sacred teacher
Climate change-related threats and damages are impacting our People and the Land	- Communities, through TEK, are observing Land changes in real time - Climate change is restricting access to our Land and cultural education
Protecting and governing our own data is crucial for preserving our stories and knowledges	- Enhancing the storage of our TEK data is necessary
We need to protect Mother Earth for future generations	- Indigenous Land management and conservation approaches are thriving in the north - Our collective responsibility is to protect our Land and Peoples from resource extraction

## Data Availability

The original contributions presented in this study are included in the article/Supplementary Materials. Further inquiries can be directed to the corresponding author.

## Supplementary Materials

The following supporting information can be downloaded at: https://www.mdpi.com/article/10.3390/ijerph23060716/s1, File S1: Interview Guide; File S2: Verbal consent form; File S3: TEK paper_SRQR_Checklist.
