# Supplementary material for: Sitting by the Fire: Dene Perspectives on Indigenous Traditional Ecological Knowledges, Land Stewardship, and Community Wellbeing
Source: Int J Environ Res Public Health. 2026 May 27;23(6):716. doi: 10.3390/ijerph23060716 (PMC13300017; doi:10.3390/ijerph23060716)
Supplement: Supplementary file 1 [file ijerph-23-00716-s001.zip › S1 Interview Guide.pdf]

INTERVIEW GUIDE

Name of researcher: ____________________________________
Interview Date/time: ____________________________________
Method of interview: ____________________________________

Welcome and Introduction

Today I will be asking you about protection of traditional ecological knowledge (TEK). Thank you for agreeing to participate in this interview and share your knowledge. The purpose of this interview is to gather information on ways to protect TEK. We will record the interview and take notes during the interview that will be analyzed and used later to integrate into a TEK toolkit and manuscript. No identifying information or names will be used in the toolkit and manuscript.

<u>Local community and knowledge system</u>

Question 1: Can you tell me about some ways your community stewards and protects its traditional knowledge systems?

Additional prompts:

a. Where do people usually share traditional ecological knowledge in your community?
b. How do people usually share traditional ecological knowledge in your community?
c. When do people usually share traditional ecological knowledge in your community?

Question 2: Do you have any stories to share on the role of traditional ecological knowledge in your community?

Question 3: What do you think affects the way traditional ecological knowledge is shared in your community?

Additional prompts:

a. Have changes in land and environment affected traditional ecological knowledge?
b. How have these changes affected traditional ecological knowledge?

<u>Protecting Traditional Knowledge</u>

Question 4: What do you think are good ways to protect traditional ecological knowledge in your community?

Question 5: What are some barriers to protecting traditional ecological knowledge in your community you think could be improved?

Question 6: Does your community have adequate processes to store and protect traditional ecological knowledge? If so, can you share more about these processes?

Future Directions

Question 7: What do you think your community can do to ensure traditional ecological knowledge is protected in the future?

Question 8: How do you think your community can continue to adapt to changes in the environment to protect traditional ecological knowledge?

Question 9: Is there anything else you would like to add or comment on in regard to anything else we have discussed today, or anything you would like to add that was not asked?

Closing and Thank You

Thank you for participating in this interview and sharing your thoughts and knowledge. After our research team has finished conducting all the interviews, we will analyze the information and the anonymized results will be incorporated into a TEK toolkit. If you have any questions about your rights as a research participant or the conduct of this study, you may contact The Office of Human Research Ethics (519) 661-3036, 1-844-720-9816, or email: ethics@uwo.ca. This office oversees the ethical conduct of research studies and is not part of the study team. Everything that you discuss will be kept confidential. If you have questions about this research study please contact Nicole Redvers at (519) 661-2111 ext. 86673 or email: nredvers@uwo.ca.
