# Supplementary material for: Sitting by the Fire: Dene Perspectives on Indigenous Traditional Ecological Knowledges, Land Stewardship, and Community Wellbeing
Source: Int J Environ Res Public Health. 2026 May 27;23(6):716. doi: 10.3390/ijerph23060716 (PMC13300017; doi:10.3390/ijerph23060716)
Supplement: Supplementary file 1 [file ijerph-23-00716-s001.zip › S2 Verbal consent form.pdf]

Western UNIVERSITY · CANADA

CONSENT TO PARTICIPATE IN A RESEARCH STUDY

Study Title: Protecting Indigenous Traditional Ecological Knowledges amidst the Climate Change Crisis**:** Biodiversity and Wellbeing Scan

Principle Investigator
Nicole Redvers, ND, MPH, Associate Professor
Western University
nredvers@uwo.ca

Co-Investigator
Danya Carroll, PhD, MPH, Postdoctoral Associate
Western University
dcarro4@uwo.ca

Introduction

You are being invited to participate in this interview about protecting Indigenous traditional ecological knowledges (ITEK) because you are an Indigenous adult over 18 years old with lived experiences and knowledge that can contribute to a toolkit we are developing to further address this topic.

Background/Purpose

Currently, many Indigenous Peoples face challenges in preserving and protecting their ITEKs as a result of many factors including climate change. There is an important need to promote capacity and support Indigenous communities in ensuring their available ITEKs are secured and protected for generations to come. Our team is working to develop a toolkit that can be shared with and support Indigenous communities through this process. We aim to conduct interviews with participants that can inform the toolkit.

Study Design

Procedures: What You Are Being Asked to Do

We will conduct in-person and virtual interviews with Indigenous participants from June 2024 until January 2025 in Canada and the United States. It is expected that the full research project will be for one year. There will be a one-time interview during your participation in this study and the interview will take approximately 60 minutes. The interview will be audio recorded if it is done in-person. If the interview is done virtually it will be audio recorded via Zoom. The consent process will be available to do in advance of the interview.

Risks:

There are no risks in participating in this research beyond those experienced in everyday life.

Benefits:

- You might learn more about yourself and your community by participating in this interview.
- Your participation may help to inform the ITEK toolkit.
- Your participation may inform strategies of addressing protection of ITEKs in Indigenous communities.
- The study also seeks to promote collective benefits by promoting Indigenous data sovereignty and the protection of health and wellbeing-related ITEK for years to come in Indigenous communities. Anticipated results for the proposed project include: the development of a set of community-level tools and processes for better ensuring the long-term protection of health and wellbeing-related ITEK repositories.

Statement of Confidentiality:
Deidentified information will be gathered as part of this study; therefore, your responses will be deidentified after this interview. If this research is published, no information that would identify you will be included in any publications. Quotes from participants may be used in publications and will be deidentified. We will develop an internal master list that will link your name to your responses using a study ID; this list will be kept separately from your data. All data (i.e. recordings, transcripts, consent forms) will be stored on the Western OneDrive server for seven years. Data from the interviews will be transferred from the audio recording device and transcribed by the NVivo transcription service.

Therefore, data from the interviews will also be deidentified, analyzed and stored in the NVivo software. NVivo's privacy policy can be found at this website: https://lumivero.com/legal-information/global-privacy-policy/ . Data stored on the internet is not 100% safe. Participants may also be asked by the researchers to confirm accuracy of what they shared during the interview during data analysis. Furthermore, deidentified data will be shared through debriefing presentations with stakeholder participants and/or organizations.

Delegated institutional representatives of Western University and its Non-Medical Research Ethics Board may require access to your study-related records to monitor the conduct of the research in accordance with regulatory requirements.

Reminders and Responsibilities

Costs
There are no costs required for participation in this study.

Compensation

You will be compensated $500 for your participation in this study. You will receive your compensation in-person, by mail or electronically if preferred after your participation.

Rights as a Participant

Your participation in this study is voluntary. You may decide not to be in this study. Even if you consent to participate you have the right to not answer individual questions or to withdraw from the study at any time. If you choose not to participate or to leave the study at any time it will have no effect on you.

You do not waive any legal right by consenting to this study.

If you decide to withdraw from the study, you have the right to request (e.g., by phone, in writing, etc.) withdrawal of information collected about you. If you wish to have your information removed, please let the researcher know and your information will be destroyed from our records. Although participants can withdraw, a record of participation must be kept and therefore, consent forms cannot be destroyed. Once the study has been published, we will not be able to withdraw your information.

Participation in the interview implies that you have read the information in this form and consent to participate in the research.

Right to Ask Questions

The researchers conducting this study are Nicole Redvers and Danya Carroll. You may ask any questions you have now. If you later have questions about the study please contact Principle Investigator, Nicole Redvers by email at nredvers@uwo.ca or phone at (519) 661-2111 ext. 86279 during the day. The Co-Investigator may be contacted at dcarro4@uwo.ca or (519) 661-2111 ext. 86279 during the day.

If you have any concerns, or complaints about your rights as a research participant or the conduct of this study, you may contact The Office of Human Research Ethics (519) 661-3036, 1-844-720-9816, or email: ethics@uwo.ca. This office oversees the ethical conduct of research studies and is not part of the study team. Everything that you discuss will be kept confidential.

This letter is yours to keep for future reference.

Verbal Consent for Protecting Indigenous Traditional Ecological Knowledges amidst the Climate Change Crisis Focus Group Study: Biodiversity and Wellbeing Scan

Thank you for responding to my email invitation to participate in a research study. My name is__________, and I am the PI/Co-PI __________ conducting this study on Protecting Indigenous Traditional Ecological Knowledges Scan amidst the Climate Change Crisis: Biodiversity and Wellbeing Scan**.** The research is being conducted to learn more about how TEK relating to health can be further protected in Indigenous communities including your community. Your participation will only be needed once for this interview that should last no more than 60 minutes.

The information provided will remain strictly confidential and you will not be identified by your answers. Data will be compiled as a whole and your deidentified individual responses will be tied to your name through an internal master list. All information disclosed during the interview will be kept in a secure server location on the Western University OneDrive. This interview will be audio recorded and notes will be taken. You may choose not to answer any question.

If you are happy to take part in this study and to give your oral consent, I will read out the Informed Consent Form that you have received a copy of, and would like you to answer 'yes' or 'no' to each of the questions.

Tick box if confirmed.

1.I confirm that I have read and understand the participant information sheet version '8th August 2024', and that I have had the opportunity to ask questions which have been answered to my satisfaction. ☐

2. I understand that my participation is voluntary and that I am free to withdraw at any stage without giving reasons and without my legal rights being affected. ☐

3. I understand that all my details will be kept confidential, that no personal information will appear on any reports or documents and only a unique ID number will be used. ☐

4. I understand that the interview will be audio-recorded and that the interview will be transcribed by an independent person to have a written record. ☐

5. I understand that data collected during the study may be looked at by individuals from the research team from the Western University or from regulatory authorities, where it is relevant to my taking part in this research. ☐

6. I agree to take part in this research study. ☐

________________________ ______________________

Name of Participant *Date*

________________________ __________________ ____________________________

Name of Person taking Consent (Print) *Date* *Signature*
